# Supplementary material for: Genetic Diversity of Pseudomonas syringae pv. actinidiae: Seasonal and Spatial Population Dynamics
Source: Microorganisms. 2020 Jun 20;8(6):931. doi: 10.3390/microorganisms8060931 (PMC7357126; doi:10.3390/microorganisms8060931)
Supplement: Supplementary file 1 [file microorganisms-08-00931-s001.zip › Figure S1 - Geographical localization of the four selected kiwifruit orchards in Portugal.pdf]

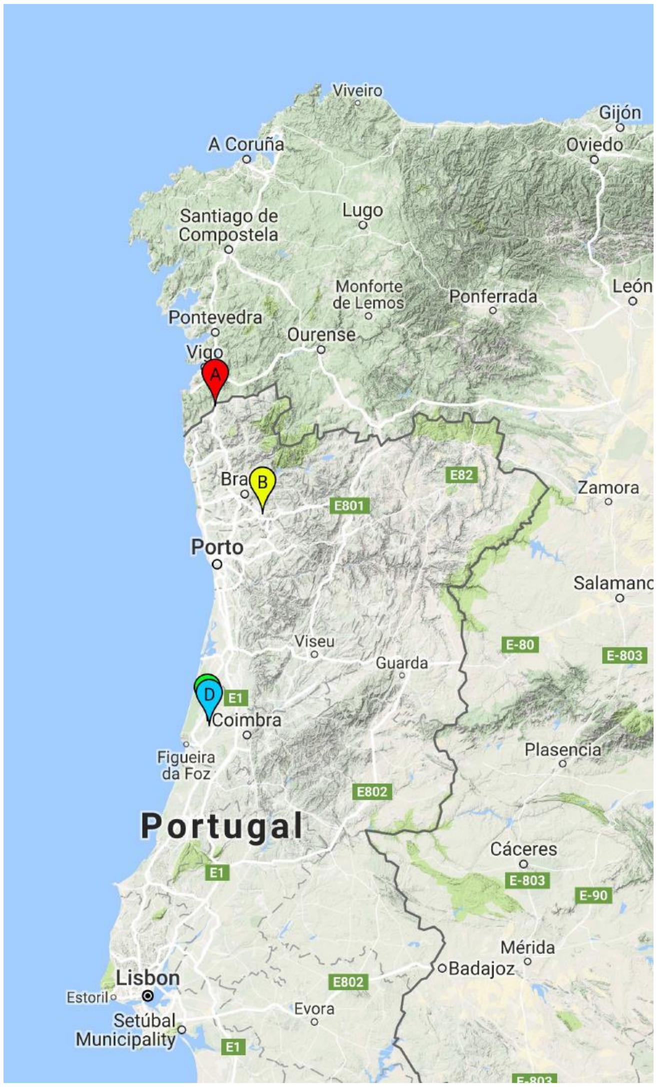

Figure S1. Geographical localization of the four selected kiwifruit orchards in Portugal. A, Valença do Minho (Viana do Castelo); B, São Esteves de Briteiros (Guimarães); C, Catarruchos (Coimbra); D, Cabeça-Alta (Coimbra). Source: https://www.google.pt/maps. Orchards A and B were 70 km away, while orchards D and E distanced by 4 km from each other. The central region orchards are at a distance of 190 km and 120 km from orchard A and B, respectively.
