# Supplementary material for: Genetic Diversity of Pseudomonas syringae pv. actinidiae: Seasonal and Spatial Population Dynamics
Source: Microorganisms. 2020 Jun 20;8(6):931. doi: 10.3390/microorganisms8060931 (PMC7357126; doi:10.3390/microorganisms8060931)
Supplement: Supplementary file 1 [file microorganisms-08-00931-s001.zip › Figure S2 -Percentage of total isolates and Psa strains recovered from kiwi orchards.pdf]

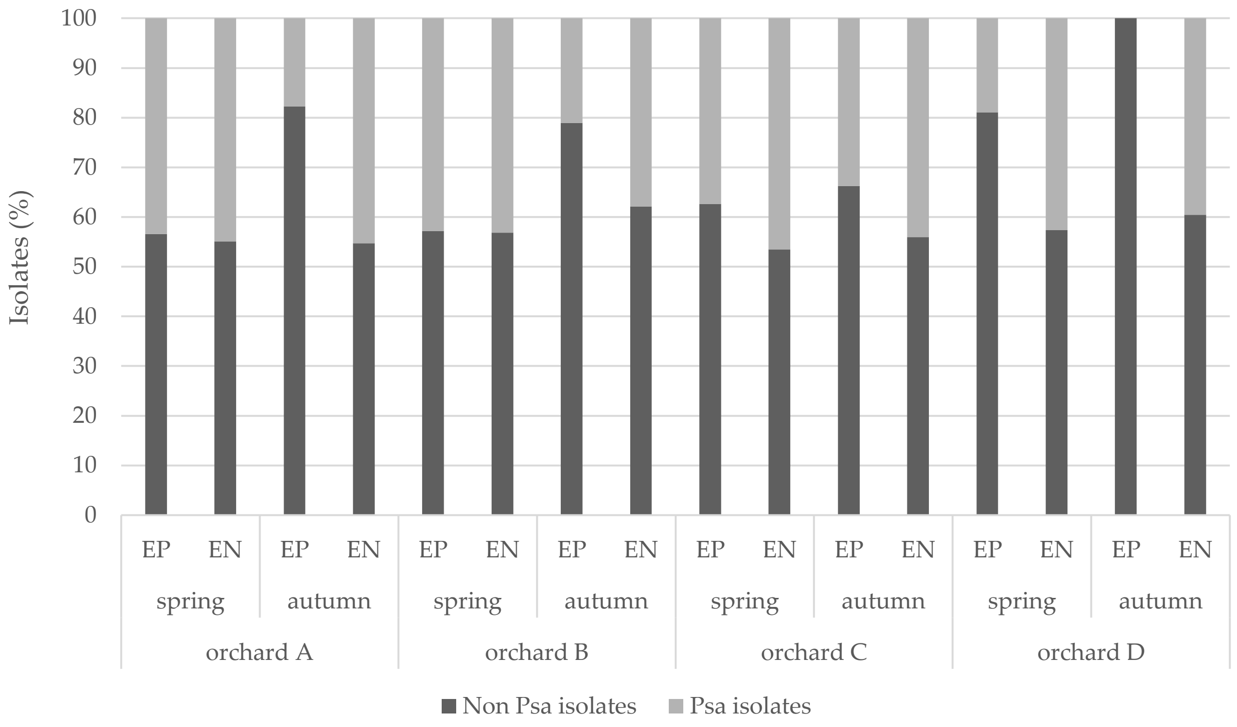

Figure S2. Percentage of total isolates and Psa strains recovered from four kiwi orchards (A to D). EP: epiphytic isolates; EN: endophytic isolates. Average value of the total number of isolates obtained from three representative plants sampled in each orchard per condition.
