# Supplementary material for: Genetic Diversity of Pseudomonas syringae pv. actinidiae: Seasonal and Spatial Population Dynamics
Source: Microorganisms. 2020 Jun 20;8(6):931. doi: 10.3390/microorganisms8060931 (PMC7357126; doi:10.3390/microorganisms8060931)
Supplement: Supplementary file 1 [file microorganisms-08-00931-s001.zip › Table S1 - Primers used for Psa identification, molecular typing and characterization.pdf]

Table S1. Primers used for Psa identification, molecular typing and characterization.

Test	ID	Primer	Amplified gene/region	Size (bp)	Primer Sequence (5'-3')	References
**Duplex-PCR**		KN-F	Genomic DNA fragment	492	CACGATACATGGGCTTATGC	Koh & Nou [42]
		KN-R			CTTTTCATCCACACACTCCG	
		AvrDdpx-F	*avrD1*	230	TTTCGGTGGTAACGTTGGCA	Galleli *et al.* [43]
		AvrDdpx-R			TTCCGCTAGGTGAAAAATGGG	
**BOX-PCR**		BOXA1R	BOX element	-	CTACGGCAAGGCGACGCTGACG	Louws *et al.* [31]
**Multiplex-PCR**	**A**	Psa F	*hopZ3*	311	CAGAGGCGCTAACGAGGAAA	Balestra *et al.* [44]
		Psa R			CGAGCATACATCAACAGGTCA	
	**B**	Europe F	PPHGI-1	733	TGGTGATCGTCTGGATGTGT	
		Europe R			ATTATGCTCCTGGCTCATGG	
	**C**	J/K F	PPHGI-1	254	AGCAACGGTGGTTTGTTTTC	
		J/K R			AAATGTTTGCCAGCCAAGTC	
	**D**	China F	PPHGI-1	609	GGAGTTCCAGCAACTGACG	
		China R			CGCTCAAGATCCTTTTCCAT	
**Phaseolotoxin**	**E**	ArgKF3	*tox–argK*	800	TCCCCCCGGGAGGAAATTCAATGAAGATTA	Templeton *et al*. [46]
		ArgKR			AACTGCAGTCAGGGGACGACTGTCTC	
**Coronatine**	**F**	CFLF	*cfl*	650	GGCGCTCCCTCGCACTT	Bereswill *et al*. [45]
		CFLR			GGTATTGGCGGGGGTGC	
**MLST**		gapA-Fps	*gapA*	675	CGCCATYCGCAACCCG	Sarkar & Guttman [47]
		gapA-Rps			CCCAYTCGTTGTCGTACCA	
		gltA-Fp	*gltA*	995	AGTTGATCATCGAGGGCGCWGCC	
		gltA-Rp			TGATCGGTTTGATCTCGCACGG	
		gltA-Fs			CCCGTCGAGCTGCCAATWCTGA	
		gltA-Rs			ATCTCGCACGGSGTRTTGAACATC	
		gyrB-Fps	*gyrB*	674	MGGCGGYAAGTTCGATGACAAYTC	
		gyrB-Rps			TRATBKCAGTCARACCTTCRCGSGC	
		rpoD-Fp	*rpoD*	812	AAGGCGARATCGAAATCGCCAAGCG	
		rpoD-Fs			GGAACWKGCGCAGGAAGTCGGCACG	
		rpoD-Rps			AAGCGTATCGAAGAAGGCATYCGTG	
