# Supplementary material for: Genetic Diversity of Pseudomonas syringae pv. actinidiae: Seasonal and Spatial Population Dynamics
Source: Microorganisms. 2020 Jun 20;8(6):931. doi: 10.3390/microorganisms8060931 (PMC7357126; doi:10.3390/microorganisms8060931)
Supplement: Supplementary file 1 [file microorganisms-08-00931-s001.zip › Table S2 - Reference strains used for the MLSA analysis.pdf]

Table S2. Reference strains used for the MLSA analysis.

Strain	Specie	Host	Isolation	Country	Locus tag				Reference
					gapA	***gltA***	***gyrB***	***rpoD***	
CFBP4909	*Pseudomonas syringae* pv. *actinidiae* (Biovar 1)	*Actinidia deliciosa*	1984	Japan (Shizuoka)	KF937408.1	KF937505.1	KF937602.1	KF937699.1	Takikawa *et al.* [5]; Ferrante & Scortichini [6]
ICMP19071	*Pseudomonas syringae* pv. *actinidiae* (Biovar 2)	*Actinidia chinensis*	1997	Korea (Jeonnam)	JN683487.1	JN683506.1	JN683468.1	JN683411.1	Koh *et al.* [16];, Chapman *et al.* [10]
T10 04758	*Pseudomonas syringae* pv. *actinidiae* (Biovar 3)	*Actinidia chinensis*	2010	New Zealand (Te Puke, Bay of Plenty)	JN683474.1	JN683493.1	JN683455.1	JN683398.1	Chapman *et al.* [10]
CFBP7811	*Pseudomonas syringae* pv. *actinidiae* (Biovar 3)	*Actinidia chinensis*	2010	New Zealand (Hawke's Bay)	KF937411.1	KF937508.1	KF937605.1	KF937702.1	Vanneste *et al.* [20]
CFBP7287	*Pseudomonas syringae* pv. *actinidiae* (Biovar 3)	*Actinidia deliciosa*	2008	Italy (Lazio)	KF937410.1	KF937507.1	KF937604.1	KF937701.1	Balestra *et al.* [1]
CFBP8047	*Pseudomonas syringae* pv. *actinidiae* (Biovar 3)	*Actinidia deliciosa*	2010	France (Rhône-Alpes)	KF937431.1	KF937544.1	KF937626.1	KF937738.1	Vanneste *et al.* (2011) [9]
MAFF212054	*Pseudomonas syringae* pv. *actinidiae* (Biovar 5)	*Actinidia chinensis*	2012	Japan (Saga)	AB842209.1	AB842197.1	AB842221.1	AB842257.1	Sawada, Miyoshi & Ide [61]
MAFF212056	*Pseudomonas syringae* pv. *actinidiae* (Biovar 5)	*Actinidia chinensis*	2012	Japan (Saga)	AB842211.1	AB842199.1	AB842223.1	AB842259.1	Sawada, Miyoshi & Ide [61]
MAFF212058	*Pseudomonas syringae* pv. *actinidiae* (Biovar 5)	*Actinidia chinensis*	2012	Japan (Saga)	AB842213.1	AB842201.1	AB842225.1	AB842261.1	Sawada, Miyoshi & Ide [61]
MAFF212061	*Pseudomonas syringae* pv. *actinidiae* (Biovar 5)	*Actinidia chinensis*	2012	Japan (Saga)	AB842216.1	AB842204.1	AB842228.1	AB842264.1	Sawada, Miyoshi & Ide [61]
MAFF212140	*Pseudomonas syringae* pv. *actinidiae* (Biovar 6)	*Actinidia deliciosa*	2015	Japan (Nagano)	LC065060.1	LC065054.1	LC065066.1	LC065084.1	Sawada *et al.* [12]
MAFF212130	*Pseudomonas syringae* pv. *actinidiae* (Biovar 6)	*Actinidia deliciosa*	2015	Japan (Nagano)	LC065055.1	LC065049.1	LC065061.1	LC065079.1	Sawada *et al.* [12]
MAFF212134	*Pseudomonas syringae* pv. *actinidiae* (Biovar 6)	*Actinidia deliciosa*	2015	Japan (Nagano)	LC065057.1	LC065051.1	LC065063.1	LC065081.1	Sawada *et al.* [12]
MAFF212138	*Pseudomonas syringae* pv. *actinidiae* (Biovar 6)	*Actinidia deliciosa*	2015	Japan (Nagano)	LC065059.1	LC065053.1	LC065065.1	KF937738.1	Sawada *et al.* [12]
CFBP7951	*Pseudomonas syringae* pv. *actinidifoliorum*	*Actinidia deliciosa*	2010	New Zeland (Bay of Plenty Tauranga)	KF937418.1	KF937520.1	KF937612.1	KF937714.1	Cunty *et al.* [21]
CFBP8041	*Pseudomonas syringae* pv. *actinidifoliorum*	*Actinidia deliciosa*	2011	France (Pays de la Loire)	KF937448.1	KF937538.1	KF937643.1	KF937732.1	Cunty *et al.* [21]
CFBP8043	*Pseudomonas syringae* pv. *actinidifoliorum*	*Actinidia deliciosa*	2011	France (Pays de la Loire)	KF937451.1	KF937540.1	KF937646.1	KF937734.1	Cunty *et al.* [21]
CFBP8045	*Pseudomonas syringae* pv. *actinidifoliorum*	*Actinidia chinensis*	1990	Australia (Western Australia)	KF937421.1	KF937542.1	KF937615.1	KF937736.1	Chapman *et al.* [10], Cunty *et al.* [21]
CFBP2212	*Pseudomonas syringae* pv. *tomato*	*Lycopersicon esculentum*	1960	United Kingdom	KF937402.1	KF937499.1	JN190421.1	JN185896.1	Gardan *et al.* [51], Gironde & Manceau [52]
