# Supplementary material for: Genetic Diversity of Pseudomonas syringae pv. actinidiae: Seasonal and Spatial Population Dynamics
Source: Microorganisms. 2020 Jun 20;8(6):931. doi: 10.3390/microorganisms8060931 (PMC7357126; doi:10.3390/microorganisms8060931)
Supplement: Supplementary file 1 [file microorganisms-08-00931-s001.zip › Table S3 - Total isolates and Psa strains recovered in this study.pdf]

Table S3. Total isolates and Psa strains recovered in this study.

Orchard	Plant	Location	Season	Nº Total Isolates	Nº Psa Isolates
A	1	Epiphytic	Spring	18	12
A	2	Epiphytic	Spring	18	13
A	3	Epiphytic	Spring	24	21
A	1	Endophytic	Spring	14	8
A	2	Endophytic	Spring	11	10
A	3	Endophytic	Spring	13	13
A	1	Epiphytic	Autumn	33	5
A	2	Epiphytic	Autumn	24	11
A	3	Epiphytic	Autumn	17	0
A	1	Endophytic	Autumn	32	29
A	2	Endophytic	Autumn	21	16
A	3	Endophytic	Autumn	22	17
B	1	Epiphytic	Spring	21	17
B	2	Epiphytic	Spring	21	16
B	3	Epiphytic	Spring	22	15
B	1	Endophytic	Spring	16	12
B	2	Endophytic	Spring	22	15
B	3	Endophytic	Spring	12	11
B	1	Epiphytic	Autumn	14	4
B	2	Epiphytic	Autumn	15	4
B	3	Epiphytic	Autumn	1	0
B	1	Endophytic	Autumn	19	17
B	2	Endophytic	Autumn	21	11
B	3	Endophytic	Autumn	19	8
C	1	Epiphytic	Spring	16	9
C	2	Epiphytic	Spring	21	15
C	3	Epiphytic	Spring	15	8
C	1	Endophytic	Spring	23	19
C	2	Endophytic	Spring	26	26
C	3	Endophytic	Spring	27	21
C	1	Epiphytic	Autumn	19	7
C	2	Epiphytic	Autumn	25	21
C	3	Epiphytic	Autumn	11	0
C	1	Endophytic	Autumn	24	17
C	2	Endophytic	Autumn	33	25
C	3	Endophytic	Autumn	23	23
D	1	Epiphytic	Spring	21	2
D	2	Epiphytic	Spring	21	3
D	3	Epiphytic	Spring	18	9
D	1	Endophytic	Spring	26	22
D	2	Endophytic	Spring	20	13
D	3	Endophytic	Spring	32	23
D	1	Epiphytic	Autumn	5	0
D	2	Epiphytic	Autumn	15	1
D	3	Epiphytic	Autumn	15	0
D	1	Endophytic	Autumn	30	24
D	2	Endophytic	Autumn	26	16
D	3	Endophytic	Autumn	28	15
**Total**				**970**	**604**
