# Supplementary material for: Genetic Diversity of Pseudomonas syringae pv. actinidiae: Seasonal and Spatial Population Dynamics
Source: Microorganisms. 2020 Jun 20;8(6):931. doi: 10.3390/microorganisms8060931 (PMC7357126; doi:10.3390/microorganisms8060931)
Supplement: Supplementary file 1 [file microorganisms-08-00931-s001.zip › Table S4 - Origin of Psa strains isolated in this study and corresponding BOX PCR profile..pdf]

Table S4. Origin of Psa strains isolated in this study, corresponding BOX PCR profile and relative abundance (%).

Orchard	Plant	Location	Season	Strain Designation	Box Profile	Relative abundance within orchard (%)
A	1	Epiphytic	Spring	KW81	-	0,6
				KW83; KW84; KW85; KW86; KW89	2	3,2
				KW97; KW133	3	1,3
				KW92; KW93; KW94; KW95	8	2,6
A	2	Epiphytic	Spring	KW342	-	0,6
				KW101	5	0,6
				KW103; KW104; KW105; KW106; KW108; KW111; KW112; KW113	9	5,2
				KW98; KW99; KW107	36	1,9
A	3	Epiphytic	Spring	KW55; KW56; KW59; KW60; KW62; KW64; KW343; KW344	-	5,2
				KW115	2	0,6
				KW67; KW68	4	1,3
				KW70	13	0,6
				KW54; KW57; KW58; KW65; KW69	23	3,2
				KW61; KW63; KW66; KW71	24	2,6
A	1	Endophytic	Spring	KW134	-	0,6
				KW130; KW135	3	1,3
				KW131;KW132	2	1,3
				KW137	6	0,6
				KW168; KW169	13	1,3
A	2	Endophytic	Spring	KW140; KW141; KW142; KW143; KW146	9	3,2
				KW147; KW148; KW150	10	1,9
				KW145; KW149	36	1,3
A	3	Endophytic	Spring	KW182	-	0,6
				KW170; KW171; KW172; KW173; KW174; KW175; KW176; KW177; KW178; KW179; KW180; KW181	10	7,7
A	1	Epiphytic	Autumn	KW1586; KW1600; KW1646; KW1659	-	2,6
				KW1641	5	0,6
A	2	Epiphytic	Autumn	KW1688	-	0,6
				KW1427; KW1429; KW1431; KW1433; KW1465; KW1619; KW1620; KW1621; KW1622; KW1682	5	6,5
A	3	Epiphytic	Autumn	-	-	0,0
A	1	Endophytic	Autumn	KW1451; KW1452; KW1453	-	1,9
				KW1356; KW1357; KW1358; KW1359; KW1360; KW1361; KW1362; KW1363; KW1364; KW1365; KW1366; KW1552; KW1553; KW1554; KW1555; KW1556; KW1557; KW1558; KW1559; KW1560; KW1561; KW1562; KW1563; KW1564; KW1565; KW1599	5	16,8
A	2	Endophytic	Autumn	KW1488	-	0,6
				KW1437; KW1440; KW1487; KW1489; KW1490; KW1491; KW1575; KW1576; KW1577; KW1579; KW1580; KW1581; KW1582; KW1680; KW1681	5	9,7
A	3	Endophytic	Autumn	KW1445	-	0,6
				KW1449; KW1480; KW1482; KW1483; KW1484; KW1485	6	3,9
				KW1447; KW1471; KW1472; KW1476; KW1477; KW1479; KW1481; KW1486; KW1583; KW1585	23	6,5
B	1	Epiphytic	Spring	KW345	-	0,8
				KW1; KW2; KW5; KW6; KW7; KW18	5	4,6
				KW3; KW4; KW17	9	2,3
				KW8; KW9; KW10; KW11; KW12; KW13; KW14	36	5,4
B	2	Epiphytic	Spring	KW80; KW121	-	1,5
				KW76; KW122	5	1,5
				KW75; KW123; KW124; KW125; KW126; KW127	9	4,6
				KW74	27	0,8
				KW73; KW77; KW78; KW79; KW128	36	3,8
B	3	Epiphytic	Spring	KW129	-	0,8
				KW154; KW156; KW157; KW158; KW159; KW160; KW161; KW165; KW167	5	6,9
				KW155; KW162; KW166	7	2,3
				KW151; KW163	13	1,5
B	1	Endophytic	Spring	KW302	-	0,8
				KW22; KW23; KW26; KW27; KW28; KW29; KW30; KW31; KW32	5	6,9
				KW24; KW25	36	1,5
B	2	Endophytic	Spring	KW49	-	0,8
				KW42; KW43; KW45; KW50	5	3,1
				KW183	7	0,8
				KW37; KW46; KW47; KW48; KW304	9	3,8
				KW38; KW39; KW40; KW44	36	3,1
B	3	Endophytic	Spring	KW184	-	0,8
				KW195	5	0,8
				KW185; KW186; KW187; KW189; KW190; KW192; KW193; KW194	7	6,2
				KW191	33	0,8
B	1	Epiphytic	Autumn	KW1468	-	0,8
				KW1413; KW1414; KW1667	5	2,3
B	2	Epiphytic	Autumn	KW1505	-	0,8
				KW1500	5	0,8
				KW1507	13	0,8
				KW1493	27	0,8
B	3	Epiphytic	Autumn	-	-	0,0
B	1	Endophytic	Autumn	KW1411; KW1609	-	1,5
				KW1412; KW1415; KW1473; KW1474; KW1475; KW1605; KW1607; KW1610; KW1611; KW1612; KW1613; KW1614; KW1616	5	10,0
				KW1606; KW1608	37	1,5
B	2	Endophytic	Autumn	KW1503; KW1504; KW1509; KW1510; KW1511; KW1512; KW1513	-	5,4
				KW1502; KW1514; KW1518; KW1519	5	3,1
B	3	Endophytic	Autumn	KW1677; KW1678; KW1679; KW1694; KW1699; KW1716; KW1717	5	5,4
				KW1618	7	0,8
C	1	Epiphytic	Spring	KW528; KW529; KW530	-	1,6
				KW522; KW524; KW525; KW526; KW527; KW533	5	3,1
C	2	Epiphytic	Spring	KW909	-	0,5
				KW539; KW540; KW542; KW544; KW545; KW546; KW547; KW548; KW549; KW550; KW870	5	5,8
				KW543; KW864; KW887	13	1,6
C	3	Epiphytic	Spring	KW553; KW600	5	1,0
				KW867; KW868; KW869; KW903; KW904; KW905	36	3,1
C	1	Endophytic	Spring	KW391; KW382; KW383; KW384; KW385; KW386; KW387; KW388; KW389; KW390; KW391; KW472; KW863; KW902; KW914	5	7,9
				KW609; KW1239; KW1310; KW1332	13	2,1
C	2	Endophytic	Spring	KW392; KW393; KW394; KW395; KW396; KW397; KW398; KW399; KW400; KW401; KW402; KW403; KW404; KW405; KW406; KW407; KW408; KW409	5	9,4
				KW536; KW537	6	1,0
				KW410; KW538	13	1,0
				KW474; KW475; KW476; KW477	36	2,1
C	3	Endophytic	Spring	KW425; KW427; KW428; KW429; KW430	5	2,6
				KW415; KW416; KW417; KW418; KW419; KW420; KW421; KW422; KW423; KW424; KW479; KW518; KW866; KW871; KW872; KW873	36	8,4
C	1	Epiphytic	Autumn	KW1776; KW1930	-	1,0
				KW1830	5	0,5
				KW1694; KW1771; KW1775; KW1993	7	2,1
C	2	Epiphytic	Autumn	KW1753; KW1754; KW1755; KW1756; KW1757; KW1763; KW1764; KW1765; KW1766; KW1767; KW1769; KW1773; KW1831; KW1832; KW1833; KW2044; KW2065; KW2066; KW2067; KW2068; KW2069	5	11,0
C	3	Epiphytic	Autumn	-	-	0,0
C	1	Endophytic	Autumn	KW1741; KW1748; KW2053; KW2054	-	2,1
				KW1744; KW1745; KW1746; KW1747; KW1780; KW1781; KW1782; KW1783; KW2055; KW2056; KW2057; KW2058; KW2075	5	6,8
C	2	Endophytic	Autumn	KW1759; KW2042; KW2094	-	1,6
				KW1760; KW1761; KW1803; KW1804; KW1805; KW1812; KW1813; KW1858; KW1859; KW1860; KW1863; KW1864; KW1977; KW1978; KW2012; KW2040; KW2041; KW2079; KW2092; KW2093; KW2095	5	11,0
				KW1762	7	0,5
C	3	Endophytic	Autumn	KW1777; KW1778; KW1779; KW1814; KW1815; KW1816; KW1817; KW1819; KW1820; KW1821; KW1822; KW1823; KW1825; KW1826; KW1827; KW1828; KW1829; KW2013; KW2071; KW2076; KW2096	5	11,0
				KW1818; KW1824	6	1,0
D	1	Epiphytic	Spring	KW1152	13	0,8
				KW517	33	0,8
D	2	Epiphytic	Spring	KW850	5	0,8
				KW779; KW780	36	1,6
D	3	Epiphytic	Spring	KW840; KW841; KW842; KW843; KW844; KW857; KW858; KW1081	5	6,3
				KW1215	13	0,8
D	1	Endophytic	Spring	KW616	-	0,0
				KW502; KW505; KW506; KW507; KW508; KW510; KW511; KW512; KW513; KW515; KW516; KW519; KW520; KW521; KW555; KW556; KW754	13	13,3
				KW503; KW504; KW509; KW514; KW518	33	3,9
D	2	Endophytic	Spring	KW765; KW766	-	1,6
				KW769; KW783; KW1013; KW1150	5	3,1
				KW771; KW772; KW773; KW774; KW777; KW778; KW781	36	5,5
D	3	Endophytic	Spring	KW802; KW803; KW804; KW805; KW806; KW807; KW808; KW809; KW811; KW812; KW813; KW815; KW816; KW817; KW853; KW854; KW855; KW856; KW894; KW895; KW1022	5	16,4
				KW1214; KW1216	13	1,6
D	1	Epiphytic	Autumn	-	-	0,0
D	2	Epiphytic	Autumn	KW1983	5	0,8
D	3	Epiphytic	Autumn	-	-	0,0
D	1	Endophytic	Autumn	KW1784; KW1786; KW1806; KW1807; KW1808; KW1809; KW1810; KW1839; KW1840; KW1841; KW1853; KW1854; KW1855; KW1856; KW1861; KW1862; KW1862; KW2011; KW2064; KW2080; KW2081; KW2082; KW2083; KW2106	5	18,8
D	2	Endophytic	Autumn	KW1787; KW1788; KW1789; KW1886; KW1887; KW1888; KW1894; KW1895; KW1896; KW1897; KW1898; KW1899; KW1906; KW2062; KW2063; KW2084	5	12,5
D	3	Endophytic	Autumn	KW1871; KW1872; KW1959; KW1960; KW1967; KW2059; KW2060; KW2061; KW2077; KW2078; KW2085; KW2086; KW2089; KW2119; KW2120	5	11,7
